# Ultrasensitive ctDNA monitoring for organ preservation in patients with locally advanced rectal cancer

**DOI:** 10.1038/s41698-025-01208-w

**Published:** 2025-12-11

**Authors:** Paolo Manca, Chin-Tung Chen, Farheen Shah, Christina Lee, Dylan Domenico, Dana Omer, Santiago Gonzalez, Ino De Bruijn, Samuel Ahuno, Walid K. Chatila, Madison Darmofal, Iman Tavassoly, Adam J. Widman, Michael F. Berger, Brian Loomis, Elli Papaemmanuil, Rona Yaeger, Asaf Zviran, Julio Garcia-Aguilar, Francisco Sanchez-Vega

**Affiliations:** 1 Department of Medicine, Memorial Sloan Kettering Cancer Center, New York, NY USA; 2 Medical Oncology Department, Fondazione IRCCS Istituto Nazionale dei Tumori, Milan, Italy; 3 Department of Surgery, Memorial Sloan Kettering Cancer Center, New York, NY USA; 4 Department of Epidemiology and Biostatistics, Memorial Sloan Kettering Cancer Center, New York, NY USA; 5 C2i Genomics Inc, New York, NY USA; 6 Department of Pathology and Laboratory Medicine, Memorial Sloan Kettering Cancer Center, New York, NY USA

**Keywords:** Cancer genomics, Rectal cancer

## Abstract

Optimal selection of patients with locally advanced rectal cancer for watch and wait (WW) and optimal management during follow-up remain challenging. We employed a primary-tumor-informed whole genome sequencing (WGS) assay to detect circulating tumor DNA (ctDNA) and estimate tumor fraction (TF) before, during and after neoadjuvant therapy. ctDNA was detected in 95% of baseline samples, and TF was a significant baseline predictor of sustained clinical complete response (scCR). High TF during or after neoadjuvant therapy was associated with lower likelihood of scCR and higher risk of relapse. Very low TF during surveillance was detected in a large proportion of patients who did not experience a recurrence, suggesting the existence of persisting low amounts of ctDNA. WGS-based ctDNA assessment thus provides high sensitivity, which is crucial for treatment de-escalation, but additional research is needed to also ensure good specificity (the trial is registered in ClinicalTrials.gov with the identifier NCT02008656).

## Introduction

Management of locally advanced rectal cancer (LARC) has shifted from the one-size-fits-all paradigm of long-course neoadjuvant chemoradiotherapy followed by total mesorectal excision (TME) and adjuvant systemic chemoth erapy to a plethora of new strategies aimed at anticipating, intensifying, deintensifying or omitting one or more of the three treatment modalities^1–5^.

Organ preservation is supported by an increasingly solid amount of evidence accumulated from retrospective case series over the years^6–10^, and recently also from prospective clinical trials. As an example, the Organ Preservation in Rectal Adenocarcinoma (OPRA) trial^11^ was a randomized phase II trial in which patients underwent total neoadjuvant therapy (TNT) in the form of induction chemotherapy followed by chemoradiotherapy (INCT-CRT) or chemoradiotherapy followed by consolidation chemotherapy (CRT-CNCT) and were subsequently offered, in case of complete or near-complete clinical response (cCR or ncCR), strict observation without rectum resection. At its most recent update, the rates of organ preservation at 5 years were 39% and 54% in the INCT-CRT and the CRT-CNCT cohorts, respectively^12^.

Watch and wait (WW) is now a standard option offered to rectal cancer patients with a cCR or ncCR to neoadjuvant therapy (NAT) in the United States^13^. Nevertheless, both patient selection and follow-up strategies are active areas of investigation for this approach. Assessment of cCR can be challenging^14^ and a substantial fraction of patients with an apparent clinical response after NAT experience regrowth during surveillance^6,12^. This happens more often in patients with ncCR and limits the organ preservation rate of this strategy. Moreover, while WW has been carried out at high-volume centers with high rectal cancer expertise and excellent resources for thorough and complete multimodal follow-up, doubts have been raised regarding broad implementation of a rectum-preserving approach, which requires important safeguards to ensure patient safety^15^. In this context, objective and precise noninvasive tools to improve patient selection based on radiological, endoscopic and clinical evaluation are urgently needed.

In parallel with the efforts to improve patient selection using endoscopy and MRI, liquid biopsy technologies have gained credibility over the past decade and are now being used for detection of minimal residual disease (MRD) in patients with LARC at some centers. Studies have demonstrated that detection of circulating tumor DNA (ctDNA) after NAT is associated with a higher risk of disease relapse and lower likelihood of a sustained clinical complete response (scCR)^16–21^. Moreover, baseline tumor fraction (TF) can be used as a proxy to estimate disease burden^22^ and has the potential to improve prediction of NAT outcomes over approaches based on routine clinical staging features alone^23^, which can be relevant for planning and optimizing the choice of the most appropriate NAT strategy. Despite this, evidence for the implementation of ctDNA in the WW strategy is scarce, with lack of adequate sensitivity remaining a critical limiting factor^24,25^.

In this context, the interest in using liquid biopsy data for MRD monitoring and the need for lowering the threshold for ctDNA detection led to the development of new assays covering the whole genome and expanding the range of detectable tumor alterations beyond point mutations and small insertions and deletions (indels)^26–29^. In this study, we evaluated the use of ctDNA assessment based on a highly sensitive, primary-tumor-informed, whole genome sequencing (WGS) assay^26^ to predict CR and disease recurrence in LARC patients from the OPRA trial.

## Results

### Patient selection

**Fig. 1:**
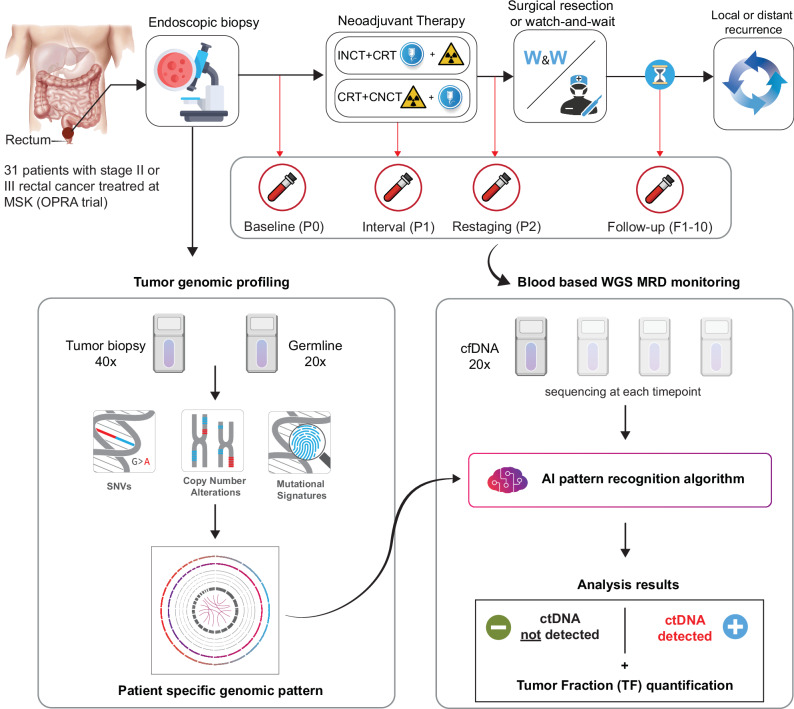
Study design and overview of computational methods for ctDNA analysis. We analyzed data from 31 LARC patients treated with NAT. Patients were randomized to receive either INCT-CRT or CRT-CNCT. Patients with a cCR or nCR were enrolled in WW, while the rest underwent TME. Longitudinal WGS data of solid tumors and matched plasma samples were analyzed for ultrasensitive detection and quantification of ctDNA. CRT-CNCT chemoradiation followed by consolidation chemotherapy, ctDNA circulating tumor DNA, cCR clinical complete response, nCR clinical near complete response, INCT-CRT induction chemotherapy followed by chemoradiation, WW watch and wait, TME total mesorectal excision.

From April 2014 until the accrual target was reached in March 2020, 324 patients were randomized in the OPRA trial. Plasma samples for ctDNA analyses were collected from 76 patients enrolled at MSKCC. Solid tumor specimens collected pre-treatment were available for 46 of those patients, and matched blood specimens were available for 32 patients. One of these 32 pairs of solid tumor and matched normal failed quality control protocols when sent for WGS, which led to the final set of 31 patients analyzed in our study (Supplementary Fig. 1). These 31 patients had plasma samples collected prospectively at baseline (P0, *n* = 21), during TNT (P1, *n* = 28), at restaging after TNT (P2, *n* = 26), and at various timepoints during follow-up (F1–F9, *n* = 102, Fig. 1). The baseline characteristic of the 31 patients included in this study did not differ significantly from the rest of the OPRA cohort, except for a slightly smaller median tumor size (4.0 vs 4.7 cm, *p* = 0.003) (Table 1); importantly, there were no significant differences between the two cohorts in terms of TME-free survival and disease-free survival (DFS) (*p* = 0.510 and *p* = 0.810, respectively).

**Table 1:** Relevant baseline clinical features in the study cohort and in the rest of the OPRA trial cohort

Features		Study cohort *n* = 31	OPRA cohort *n* = 293	*p* value
Age at diagnosis	Median (IQR)	57 (51–67)	58.5 (49.5–66.9)	0.849
Sex	Female	10 (32%)	109 (37%)	0.697
	Male	21 (68%)	246 (88%)	
Race	Asian	3 (10%)	14 (5%)	0.728
	Black or African American	1 (3%)	17 (6%)	
	White	27 (87%)	246 (88%)	
	Unknown	0	12	
Ethnicity	Hispanic or Latino	3 (10%)	15 (5%)	0.397
	Non-Hispanic	28 (90%)	277 (95%)	
	Unknown	0	1	
cT	1/2	4 (13%)	28 (10%)	0.527
	3/4	27 (87%)	265 (90%)	
cN	cN−	7 (23%)	87 (30%)	0.533
	cN+	24 (77%)	206 (70%)	
Anorectal verge distance [cm]	Median (IQR)	4.6 (3.9–5.8)	4.4 (3.0–6.6)	0.549
Primary tumor size [cm]	Median (IQR)	4.0 (3.0–4.6)	4.7 (3.8–5.7)	0.003
Extramural venous invasion	Absent	27 (87%)	185 (71%)	0.181
	Undetermined	0 (0%)	15 (5.8%)	
	Present	4 (13%)	60 (23%)	
	Unknown	0	33	
Treatment arm	INCT-CRT	16 (52%)	150 (51%)	1.000
	CRT-CNCT	15 (48%)	143 (49%)	

*P*-values are calculated using Fisher exact test for categorical variables or Wilcoxon signed-rank test for numerical variables.

*cN* clinical nodal classification, *CRT-CNCT* chemoradiation followed by consolidation. chemotherapy, *cT* clinical tumor classification, *INCT-CRT* induction chemotherapy followed by chemoradiation.

All 31 patients received TNT in the form of either INCT-CRT or CRT-CNCT. There were no differences in baseline patients and tumor characteristics between the two arms, except for a higher rate of extramural venous invasion in the INCT-CRT group (26.7% vs. 0%, *p* = 0.043) (Supplementary Table 1). All patients completed planned chemoradiotherapy cycles, while the overall completion rate of induction/consolidation chemotherapy was 94%.

**Fig. 2:**
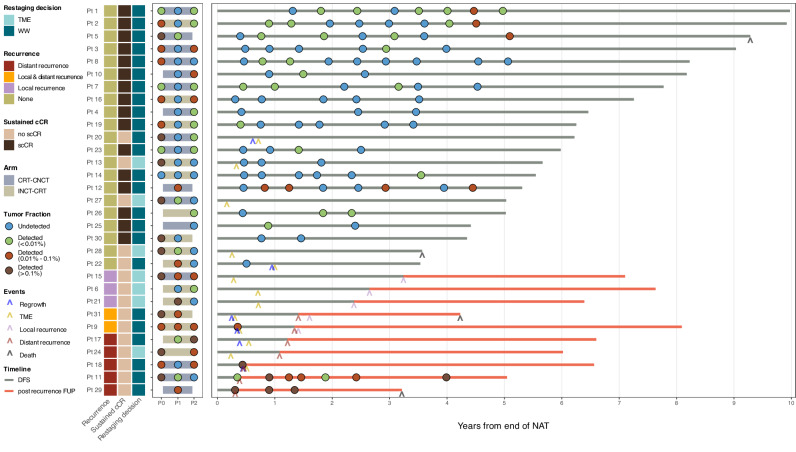
Timeline of ctDNA detection results and treatment outcomes. Relevant clinical data and results from ctDNA assessments are summarized using a swimmer plot. The circles represent the available liquid biopsies and are colored in blue, green, light brown or dark brown according to the detection of ctDNA and the respective tumor fraction. Y-axis reports anonymized patients ID. CRT-CNCT chemoradiation followed by consolidation chemotherapy, ctDNA circulating tumor DNA, scCR sustained clinical complete response, DFS disease-free survival, FUP follow-up, INCT-CRT induction chemotherapy followed by chemoradiation, WW watch and wait, TME total mesorectal excision, TF tumor fraction.

Patients were restaged after a median time of 8 weeks following completion of TNT, at which 24 patients had a cCR (*n* = 14) or nCR (*n* = 10) and were offered the option of WW per protocol (Fig. 2). Of those 24 patients offered WW, 6 (25%) developed local tumor regrowth during surveillance and required TME; 4 of them developed local recurrence, distant metastasis or both, and 2 remain without evidence of disease. The remaining 18 patients did not experience primary tumor regrowth; nevertheless, 2 patients developed distant metastases during surveillance. In total, 16 (52%) of 31 patients never developed tumor regrowth, locoregional recurrence or distant metastasis at the end of follow-up and were defined as having a scCR. By contrast, of the 7 patients initially treated with TME, one developed distant metastasis, 3 developed local recurrence and 3 remained without evidence of disease until the last available follow-up. In total, after a median follow-up of 6.52 years, ten patients experienced disease recurrence.

As in the OPRA trial, the CRT-CNCT group and the INCT-CRT group did not differ significantly in terms of DFS, overall survival, local recurrence-free survival, or distant metastasis-free survival (Supplementary Fig. 2).

### ctDNA and TF at baseline

**Fig. 3:**
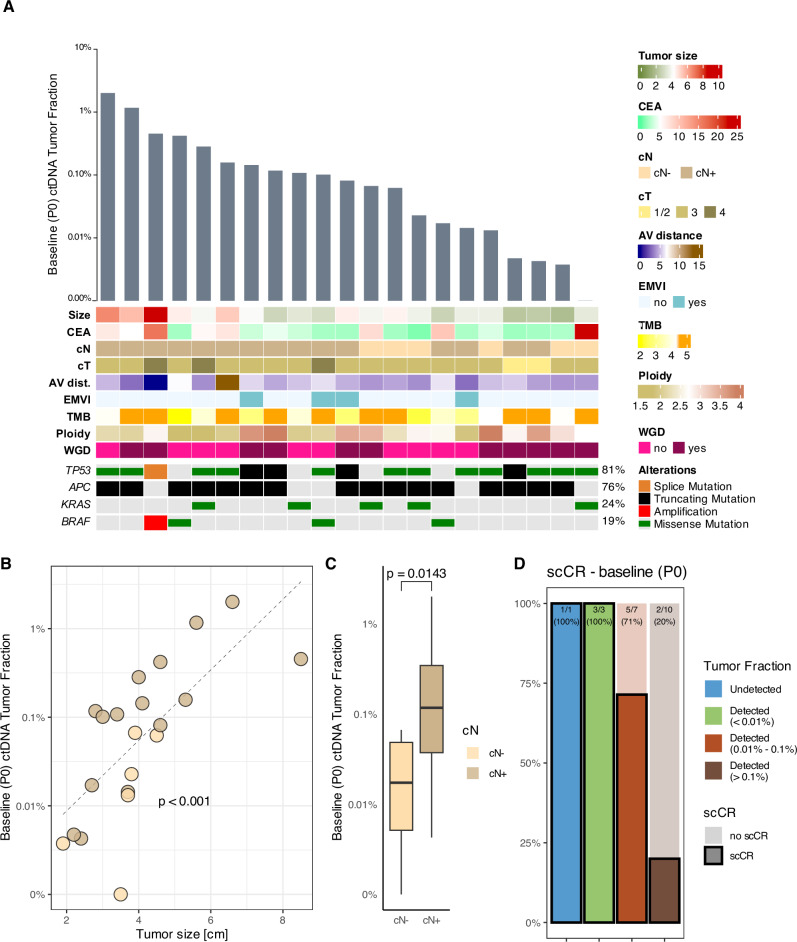
Assessment of baseline ctDNA. **A** Heatmap showing (from top to bottom): the TF of the baseline P0 liquid biopsy (before the initiation of the NAT) in log_10_ scale, relevant baseline clinical features of the patients and alterations of *TP53*, *APC*, *KRAS* and *BRAF* from primary tumor samples acquired before the initiation of the neoadjuvant treatment; each column represents a patient, columns are ordered according to the P0 TF. **B** Scatterplot showing the primary tumor size (x-axis) and the TF of the baseline P0 liquid biopsy in log_10_ scale; each dot is a patient and is colored according to the clinical N staging; the dashed line parameters were calculated using linear regression, the *p*-value was calculated using Spearman correlation; for graphic purposes, a sample with undetected ctDNA was plotted as TF = 0.001%. **C** Boxplot representing the baseline P0 liquid biopsy TF in log_10_ scale according to the radiological suspicion of lymph nodes metastasis; the *p*-value was calculated with Wilcoxon signed-rank test; for graphic purposes, a sample with undetected ctDNA was plotted as TF = 0.001%. **D** Proportion of patients with scCR depending on the detection of ctDNA and the respective TF at the P0 timepoint. AV distance distance from anal verge, cN clinical nodal classification, cT clinical tumor classification, ctDNA circulating tumor DNA, scCR sustained clinical complete response, EMVI Extramural vascular invasion, T size primary tumor size at baseline according to radiological measurement, scCR sustained clinical complete response, TMB tumor mutational burden, TF tumor fraction, WGD whole genome doubling.

Baseline plasma samples (P0 timepoint) were available for 21 of the 31 patients (Fig. 2); ctDNA was detected in plasma samples of 20 patients (95%; 95% confidence interval, 74–100%), with a median TF of 0.081% (interquartile range [IQR], 0.014–0.158%) (Fig. 3A). TF detected at P0 was positively correlated with tumor size (ρ = 0.76, *P* < 0.001) (Fig. 3B) and with the presence of locoregional nodal metastasis at baseline MRI (median, 0.12% vs 0.018%; *P* = 0.014) (Fig. 3C). No other associations between baseline TF and clinical or genomic features were statistically significant (Supplementary Table 2).

Next, we investigated whether the disease burden represented by baseline TF was predictive of treatment outcomes. Using exploratory TF cutoffs of 0.1% and 0.01%, we found that all 4 patients (100%) with a TF < 0.01% had a scCR, compared to 5 of 7 patients (71%) with a TF between 0.01 and 0.1%, and in 2 of 10 (20%) patients with a TF > 0.1% (Fig. 3D). Baseline TF modeled as a continuous numerical variable was significantly associated with scCR in univariate logistic regression (*P* = 0.048) (Supplementary Table 3), with an AUC for scCR prediction of 0.809 (95% confidence interval, 0.609–1.000) (Supplementary Fig. 3A).

### ctDNA and TF during NAT and at restaging after NAT

We next investigated ctDNA assessment at time points P1 (right after the completion of the first component of NAT) and P2 (restaging after NAT). The median time between the end of the first component of NAT (INCT in the INCT-CRT group and CRT in the CRT-CNCT group) and P1 was 3.8 weeks (IQR, 3.0–4.5 weeks). The median time from the end of NAT to P2 was 8.0 weeks (IQR, 6.1–9.3 weeks). All patients had a P1 or a P2 liquid biopsy available, or both (Fig. 2). ctDNA was detected in 12 out of 28 patients (43%) at P1 and 17 out 26 patients (65%) at P2, with no statistically significant difference between CRT-CNCT patients and INCT-CRT patients.

**Fig. 4:**
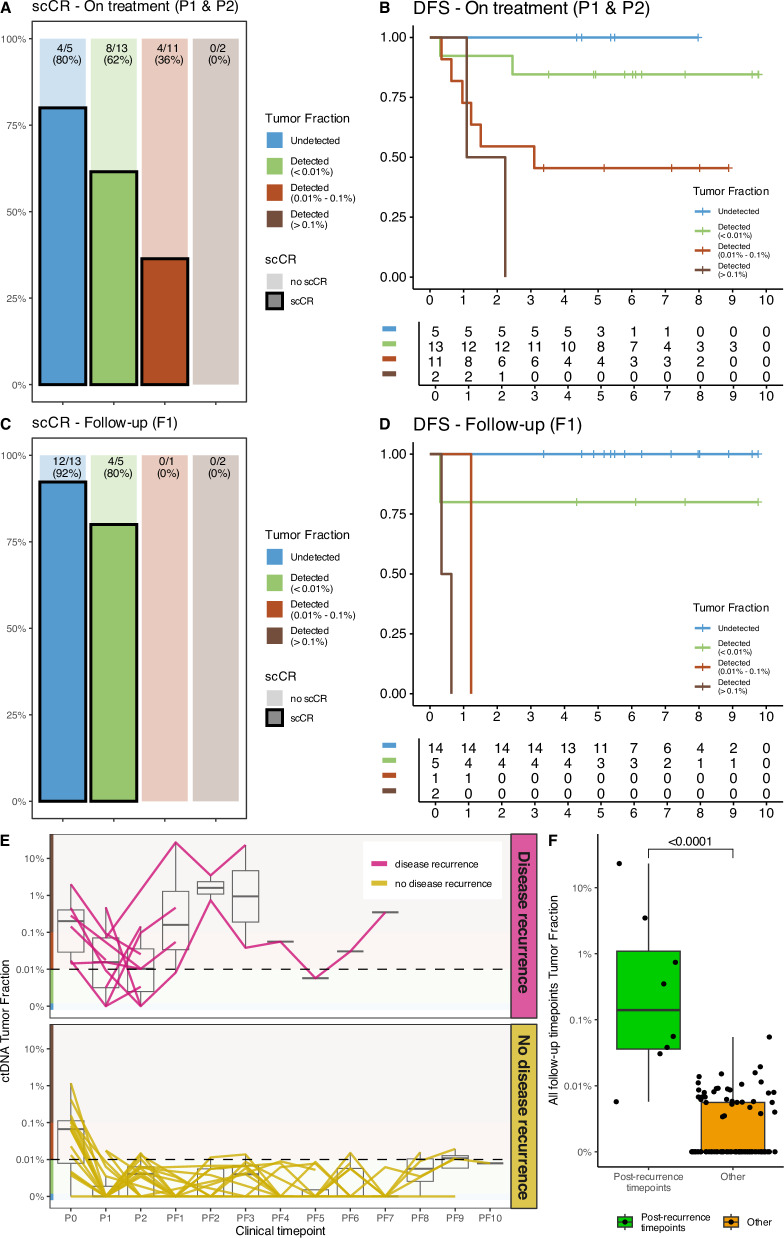
ctDNA assessment during the TNT and the follow-up. **A** Proportion of patients with scCR depending on the detection of ctDNA and the respective TF at the P1 and P2 timepoints; the highest available value between P1 and P2 was used for this purpose. **B** Kaplan–Meier curves depicting DFS depending on the detection of ctDNA and the respective TF at the P1 and P2 timepoints; DFS was measured starting from the moment of the liquid biopsy blood withdrawal; the highest available value between P1 and P2 was used for this purpose; **C** Proportion of patients with scCR depending on the detection of ctDNA and the respective TF at the F1 timepoint; for this panel, only patients proposed for WW observation were considered; **D** Kaplan–Meier curves depicting DFS depending on the detection of ctDNA and the respective TF at the F1 timepoint; DFS was measured starting from the moment of the liquid biopsy blood withdrawal; **E** TF across all available timepoints; each line is a patient, colored depending on the presence of disease relapse; the dashed line represent the 0.01% cut-off; **F** TF of all follow-up timepoints depending on the disease relapse timing; the p-value was calculated using Wilcoxon signed-rank test; for graphic purposes, a sample with undetected ctDNA was plotted as TF = 0.001%. ctDNA circulating tumor DNA, DFS disease-free survival, scCR sustained clinical complete response.

To identify potential patterns of delayed response or early resistance, we stratified the cohort according to the higher of the P1 and P2 TFs, and we used the exploratory cutoffs of 0.01% and 0.1% to compare their response rates. Four (80%) of the 5 patients with no TF detected at P1 and P2 had a scCR, compared to 8 (62%) of the 13 patients with TF < 0.01%, 4 (36%) of the 11 patients with TF between 0.01% and 0.1%, and neither of the 2 patients with TF ≥ 0.1% (Fig. 4A). The higher of the P1 and P2 TFs, modeled as a continuous variable, was linearly associated with a lower likelihood of scCR (OR, 0.10; 95% confidence interval, 0.01-0.48); logistic regression *P* = 0.018) (Supplementary Table 4 and Supplementary Fig. 3B) and a poorer DFS (HR, 4.20; 95% confidence interval, 1.87–9.43; Cox regression *P* < 0.001; Harrell’s C-index, 0.792) (Fig. 4B and Supplementary Table 5).

Detectable ctDNA at P1 was associated with a lower likelihood of scCR (16.7% vs 75.0%; *P* = 0.006) and a higher risk of disease relapse (hazard ratio [HR], 6.47; 95% confidence interval, 1.33–31.41; *P* = 0.020) (Supplementary Fig. 4A, B). However, detectable ctDNA at P2 was not associated with either scCR likelihood in WW candidates (71.4% vs 60.0%; *P* = 1.000) or risk of disease relapse (HR, 1.63; 95% confidence interval, 0.61–8.07; *P* = 0.552) (Supplementary Fig 4C, D).

In exploring the counterintuitive finding of an association at P1 and no association at P2, we observed that 12 (80%) of the 15 patients with no detectable ctDNA at P1 had detectable ctDNA at P2 (Supplementary Fig. 5A) and that only 2 (17%) of the 12 experienced disease relapse. Compared with the other patients, the 12 P1-negative P2-positive patients had lower TFs at baseline (median 0.017% vs 0.13%, *P* = 0.018) (Supplementary Fig. 5B), possibly suggesting that a phenotype with lower ctDNA shedding might have affected the specificity of the assay at P2. Low specificity was also suggested by the association of detectable ctDNA at P2 with higher risk of disease recurrence when the TF threshold for presence of MRD was set at 0.01% (HR, 5.09; 95% confidence interval, 1.20–21.55; *P* = 0.027) (Supplementary Fig. 5C).

### ctDNA and TF during follow-up

At least one follow-up liquid biopsy (*n* = 102) was available for 22 patients. The median time between P2 and the first follow-up (F1) ctDNA assessment was 14.0 weeks (IQR, 12.3–18.1 weeks). The mean number of liquid biopsies per patient was 4.9 (range 1–9). Half of the patients had samples available beyond 3 years from the end of NAT.

High TF at F1, modeled as a continuous variable, was associated with higher risk of disease relapse (Cox regression *P* = 0.009), which occurred in all 3 patients with TF > 0.01% but in only 1 of 5 patients with TF < 0.01% and in none of the 14 patients with undetectable ctDNA (Fig. 4C). We noted a trend for negative association between TF at F1 and scCR (logistic regression *P* = 0.062) (Fig. 4D). An increase in TF between P2 and F1 was associated with poorer DFS (log rank *P* = 0.002) (Supplementary Fig. 6A) and a trend toward a lower likelihood of scCR (40.0% vs 91.6%; *P* = 0.053) (Supplementary Fig. 6B).

With inclusion of follow-up liquid biopsies at timepoints after F1, the sensitivity of detection of ctDNA at any time point for predicting disease recurrence was 100%, but specificity was 27.8%, resulting in a positive predictive value of 23.5%. However, the median TF of follow-up liquid biopsies performed after tumor recurrence was significantly higher than the median TF of follow-up biopsies performed in the absence of tumor recurrence (*P* < 0.001) (Fig. 4E, F).

In 4 patients undergoing WW who did not experience neither disease recurrence nor local regrowth, the last ctDNA TF > 0.1% was obtained more than 4 years after the end of NAT. Intriguingly, in selected patients with follow-up data available at multiple timepoints, we observed a paired fluctuation of TF and carcinoembryonic antigen (CEA), both in patients who did not experience a disease relapse and had low TF and clinically nonrelevant CEA values (patients 2, 5 and 19 in Supplementary Fig. 7) and in patients who later developed metastatic disease and had the highest CEA and TF (patients 11 and 29 in Supplementary Fig. 7).

### Additional insights from WGS of solid tissue specimens

**Fig. 5:**
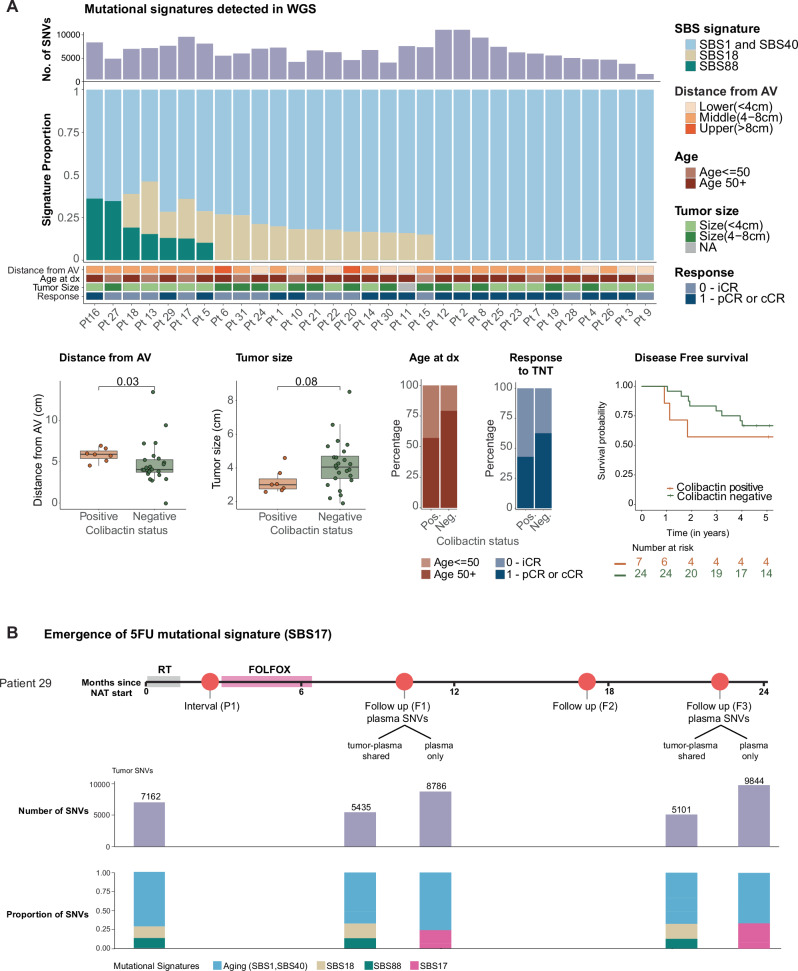
Baseline and longitudinal analyses of mutational signatures. **A** Number of SNVs and mutational signatures detected from tumor tissue WGS; **B** Emergence of FOLFOX treatment associated mutational signature in blood based WGS in patient #29.

Besides providing information for ultrasensitive detection of ctDNA, WGS from solid tissue offers additional insights into tumor etiology and tumor evolution throughout treatment that can be translationally relevant. As an example, we used these data to analyze mutational signatures from the COSMIC catalog^30^ (Fig. 5A, B). After removing potential FFPE-related sequencing artifacts that could introduce noise in our analysis, we identified the presence of three main signatures within this cohort. The mitotic clock, age-related SBS1/SBS40 signatures were consistently detected across all samples. SBS18, which has been associated with damage by reactive oxygen species, was detected in 17/31 (54%) cases. Finally, a colibactin (Escherichia coli exposure)-associated signature (SBS88) was identified in 7/31 (23%) of the specimens collected at baseline (Fig. 5A). This frequency of colibactin positive cases is consistent with data from a larger case series^31^. Even though our sample size was too small to draw robust statistical conclusions, colibactin positive tumors tended to be smaller and located further from the anal verge. Patients with colibactin positive tumors also exhibited lower rates of pathological or clinical complete responses to TNT and worse DFS (although differences were not statistically significant, Fig. 5A).

Longitudinal analysis of WGS data from solid tumors and matched ctDNA samples also revealed the emergence of a treatment-related signature (SBS17b) after NAT, which was detected in plasma of patient 29 (Fig. 5B). Of note, this was the only patient in the study with a sufficiently high detected TF to enable de novo mutation calling from plasma samples at F1 and F3. The analyses of those mutations demonstrated a high-concordance with the set of mutations that were initially identified in the reference solid tumor, but also the existence of an additional set of new mutations that were private to the plasma samples. The SBS17b signature detected among these novel mutations is consistent with the systemic chemotherapy treatment that the patient had undergone during NAT.

## Discussion

In recent years, several studies have demonstrated the clinical utility of WW for patients with rectal cancer^6–10^, and this has been further validated by the results from the OPRA trial^11^. The findings of our translational study, aimed at improving both the selection of rectal cancer patients for WW and follow-up monitoring, indicate that primary-tumor-informed analysis of liquid biopsies can be used to measure disease burden and might be useful to monitor NAT efficacy, with the caveat of low specificity for detection of MRD and prediction of scCR.

Accurate prediction of the expected response to pre-operative treatment is key for the clinical management of LARC patients. For patients who undergo TNT and WW, our results confirm that high disease burden at baseline is associated with lower likelihood of scCR and that consideration of TF can help improve prognostic stratification. The 95% ctDNA positivity at baseline was higher than the 75–84% reported in other studies^16–21^, which were not able to stratify outcomes according to baseline ctDNA levels. Given the similarity between the cohorts, the lower rate of ctDNA detection in the other studies can probably be attributed to lower sensitivity of the assays used in those studies rather than differences in patient characteristics. Clinical trials exploring new modalities for treatment intensification or deintensification should further investigate the predictive value of baseline TF (rather than just ctDNA positivity) as a translational biomarker, using high-sensitivity techniques such as WGS.

The 43% ctDNA positivity at P1 (completion of the first component of NAT) was higher than the 8–23% ctDNA positivity after neoadjuvant CRT in the previous series^16–21^. Similarly to our results, those studies found that ctDNA positivity at completion of neoadjuvant CRT was associated with disease recurrence (HR, 9.11 [meta-analysis of the six studies^32^] vs 6.47 in our study [for ctDNA detection at completion of the first component of NAT]). Our data confirm the prognostic value of ctDNA detection at completion of either neoadjuvant CRT or neoadjuvant INCT and this could be used to refine the criterion of 20% tumor reduction that was used for omitting CRT after INCT in the clinical trial reported by Schrag et al.^5^ – although we are underpowered to have an effective comparisons of the two treatment arms due to our limited sample size.

The high rate of ctDNA positivity at post-NAT restaging (P2) and in follow-up, mostly in patients with low TF and no subsequent disease recurrence, poses some concerns for the clinical implementation of ultra-sensitive ctDNA assays to predict WW outcomes in rectal cancer. It is possible that a high baseline level of nontumoral circulating DNA may have lowered the specificity of the assay, with shedding of CEA and nontumoral DNA from normal colonic cells^33^ causing the paired fluctuation of TF and CEA. However, this is unlikely due to the tumor-informed nature of our pipeline, which incorporates the molecular profile of the primary tumor into the detection method. Alternatively, the low TF may actually reflect shedding of tumoral cells at a level insufficient to overcome immune control and cause regrowth. In the context of WW, it may not be possible for ctDNA detection to reach the high specificity for disease recurrence observed in other settings at the post-surgical^34–36^ or at the landmark timepoints^37^. However, Amatu et al.^24^ recently reported that post-TNT targeted sequencing informed by primary-tumor mutations had high specificity but low sensitivity and low overall accuracy. The WGS-based ctDNA assay used in our study does not have low sensitivity (a major concern with liquid biopsy-based assessments of MRD^38,39^), but our results suggest that isolated detection of ctDNA in WW patients with a highly sensitive primary-tumor-informed assay is not a sufficient predictor of regrowth and should be combined with dynamic monitoring of TF.

The WGS data from solid tumors and matched plasma samples generated as part of our analysis pipeline provides biological insights that go beyond MRD detection and can have translational relevance. As proof-of-concept, we robustly identified colibactin signatures in almost one fourth of the patients in our cohort. Colibactin is a genotoxin produced by some strains of E. coli that harbor the polyketide synthase (pks) genomic island and are often found in the human gut^40^. Previous studies have shown that colibactin can cause double-strand breaks and mutations in host cells^41^, contributing to the pathogenesis of colorectal cancer^42,43^. Even though our sample size is limited, our results suggest that these signatures may be associated with differences in clinical outcomes, and could therefore potentially be used as future biomarkers for patient stratification.

The main limitation of our study is the relatively small sample size, which prevented analysis of differences between the INCT-CRT and CRT-CNCT groups and which warrants caution in drawing conclusions from the data. In particular, the lack of statistical significance in some of our comparisons might be explained by our limited statistical power and some of these results should be revisited in future, larger cohorts. In addition to this, missingness of plasma samples that were not collected or failed QC at some specific timepoints, particularly for patients who underwent resection of the primary tumor, prevented meaningful comparison across follow-up measurements and make it difficult to generalize some of our findings, including the high assay sensitivity at baseline and the relevance of TF changes between P0, P1 and P2. The TF thresholds that we have used in this manuscript are purely exploratory, and they were used to illustrate the usefulness of stratifying patients based on TF levels that go beyond the simple binary classification of ctDNA presence or absence. Before any clinical utility of these thresholds can be claimed, they should be optimized and validated using larger, independent cohorts. Notwithstanding these limitations, our study is the first to investigate the use of WGS-based, ultra-sensitive longitudinal analyses of ctDNA in LARC patients undergoing WW and to describe both its strengths and technical challenges.

In conclusion, our results demonstrate that WGS approaches for tumor-informed ctDNA analysis can achieve high sensitivity and hold promise for treatment planning, prognostication and monitoring in LARC patients treated with NAT. However, additional research is needed in order to guarantee adequate specificity for MRD detection and early relapse prediction. Addressing this question will be critical in order to assess the clinical utility of liquid biopsies for monitoring LARC patients undergoing non-operative management.

## Methods

### Patients’ and data collection

This preplanned secondary retrospective analysis used data from a subgroup of patients from the OPRA trial^11^, a phase II randomized study evaluating DFS of patients with stage II or III rectal cancer who underwent TNT and selective WW in case of a cCR or a nCR. Patients with LARC were randomized to undergo either CRT-CNCT or INCT-CRT. INCT and CNCT consisted, at the oncologist’s discretion, of either eight 2-week cycles of infusional fluorouracil, leucovorin, and oxaliplatin chemotherapy (FOLFOX) or five 3-week cycles of capecitabine and oxaliplatin (CAPOX); chemoradiotherapy consisted of a long course radiotherapy with concomitant oral or infusional fluoropyrimidine treatment as chosen by the investigators. WW candidates underwent a closer follow-up monitoring with pelvic MRI, CT of the chest and abdomen and colonoscopy every 4 months. The last follow-up update was in April 2023. The data collected for this study were stored in a secured database at MSK.

We analyzed specimens from all OPRA patients treated at MSK with available pre-treatment formalin-fixed, paraffin-embedded (FFPE) primary tumor tissue samples and available plasma samples collected at baseline and throughout the course of treatment. Given the exploratory nature of the study, no formal sample size calculation was performed.

### Blood samples

For each patient, plasma was collected before the start of NAT (timepoint P0), right after the completion of INCT in the INCT-CRT group or completion of CRT in the CRT-CNCT group (P1), 8 ± 4 weeks after the end of NAT (P2), and at follow-up visits (F1, F2, etc.) (Fig. 1).

### DNA isolation and sequencing

Pathologist-guided microdissection was carried out to enrich tumoral DNA content from FFPE samples of primary tumors of eligible patients. DNA was extracted using either the AllPrep DNA/RNA FFPE Kit (Qiagen) or the Qiagen DNeasy Tissue Kit (Qiagen). Plasma samples were collected in BD Vacutainer K2-EDTA tubes and stored at −80 °C; circulating DNA was subsequently extracted by centrifugation. Both plasma-derived DNA and primary-tumor-derived DNA were sequenced using the NovaSeq platform (Illumina), employing paired-end sequencing with a read length of 150 bp.

### Detection of ctDNA and estimation of TF

ctDNA was assessed using a primary-tumor-informed assay based on WGS, as previously described^26^. Specifically, we used the C2i Genomics platform, which has been validated and used in previously published studies^28,29^. The algorithm uses the complete set of somatic alterations (single-nucleotide variants [SNVs], small indels and copy number alterations) found in a patient’s tumor sample to generate a patient-specific tumor signature, which is then applied to detect ctDNA in cell-free DNA from blood samples and to estimate TF. To address WGS artifacts, the signatures were refined by excluding alterations detected in at least 3 of 45 cell-free DNA samples from healthy individuals. Furthermore, suppression of cell-free DNA read-level error was implemented to eliminate SNV-like artifacts by assessing concordance between overlapping read-pair sequences.

### Analyses of mutational signatures in solid tumors and plasma samples

Cancer mutational signature analysis was performed both in solid tumor samples and cfDNA plasma samples (Fig. 5A, B). Plasma specimens with a TF above 10% were selected for de novo calling of SNVs and CNVs. SNV and INDEL results from the plasma samples were compared with results from the solid tumor samples to determine shared, tumor-unique, and plasma-unique alterations.

Sigprofiler v1.1.20^44^ was used to extract SNV and INDEL signatures in a three-step workflow: (I) De-novo extraction of signatures for potential new signatures in the cohort. (II) Fitting of selected signatures from COSMIC v3.3^45^ for cancer signatures and artifact signatures used for the detection of spurious deviations^30^. (III) A final fitting using cancer-type exclusive signatures and the manual inclusion of select signatures determined from steps (I) and (II), that corresponds to the final set of signatures presented in the results.

### Outcomes and statistical analysis

DFS was calculated from randomization until local or distant disease recurrence; as per the original protocol^12^, the occurrence of an operable cancer regrowth in patients followed-up with WW intent was not considered a treatment failure. Overall survival (OS), local recurrence-free survival and distant recurrence-free survival were calculated from the date of randomization until, respectively, death, local recurrence or distant recurrence. Presence of cCR/nCR was assessed at the time of post-NAT restaging according to the radiologic, endoscopic and clinical criteria of the original study. For this study, patients with cCR/nCR who did not develop later tumor regrowth or disease recurrence were defined as having a scCR.

Right-censored data were modeled using univariate Cox regression. For ctDNA-based predictors, left truncation was employed in order to consider the date of the blood draw and not the randomization date as the start of observation. Log rank was used despite Cox regression in cases where a *P* value could not be computed due to the absence of events in one of the two groups. Logistic regression was used to model binomial outcomes. When TF was used as a numerical predictor in Cox and logistic regressions, log_10_ transformation was applied, and TF of 0 was entered as 0.001%. The Fisher exact test and the Wilcoxon signed-rank test were used to compare categorical and numerical variables, respectively. Correlation between numerical variables was assessed with the Spearman ρ coefficient.

### Ethics approval and consent to participate

This study was conducted in accordance with the principles of the declaration of Helsinki. All subjects provided informed consent as specified in the study protocol. The MSKCC Institutional Review Board/Privacy Board (IRB/PB) approved the study protocol and the respective relevant documentation prior to its implementation (IRB number: 13-213 A23).

## Supplementary information

Supplementary_Materials.

## Data Availability

De-identified clinical data comprising sample IDs, sequencing results and tumor fraction assessments and time will be made available upon request to the corresponding author. The raw sequencing data can also be requested for research use, but sharing may require execution of a data transfer agreement with MSK. Code for generating the analyses and plots from this manuscript can be shared upon request to the corresponding author.

## References

[1] Fokas, E. et al. Randomized Phase II Trial of Chemoradiotherapy Plus Induction or Consolidation Chemotherapy as Total Neoadjuvant Therapy for Locally Advanced Rectal Cancer: CAO/ARO/AIO-12. *J. Clin. Oncol.* **37**, 3212–3222 (2019). 31150315 10.1200/JCO.19.00308

[2] Jin, J. et al. Multicenter, Randomized, Phase III Trial of Short-Term Radiotherapy Plus Chemotherapy Versus Long-Term Chemoradiotherapy in Locally Advanced Rectal Cancer (STELLAR). *J. Clin. Oncol.* **40**, 1681–1692 (2022). 35263150 10.1200/JCO.21.01667 PMC9113208

[3] Bahadoer, R. R. et al. Short-course radiotherapy followed by chemotherapy before total mesorectal excision (TME) versus preoperative chemoradiotherapy, TME, and optional adjuvant chemotherapy in locally advanced rectal cancer (RAPIDO): a randomised, open-label, phase 3 trial. *Lancet Oncol.* **22**, 29–42 (2021). 33301740 10.1016/S1470-2045(20)30555-6

[4] Conroy, T. et al. Neoadjuvant chemotherapy with FOLFIRINOX and preoperative chemoradiotherapy for patients with locally advanced rectal cancer (UNICANCER-PRODIGE 23): a multicentre, randomised, open-label, phase 3 trial. *Lancet Oncol.* **22**, 702–715 (2021). 33862000 10.1016/S1470-2045(21)00079-6

[5] Schrag, D. et al. Preoperative Treatment of Locally Advanced Rectal Cancer. *N. Engl. J. Med.* **389**, 322–334 (2023). 37272534 10.1056/NEJMoa2303269 PMC10775881

[6] van der Valk, M. J. M. et al. Long-term outcomes of clinical complete responders after neoadjuvant treatment for rectal cancer in the International Watch & Wait Database (IWWD): an international multicentre registry study. *Lancet* **391**, 2537–2545 (2018). 29976470 10.1016/S0140-6736(18)31078-X

[7] Renehan, A. G. et al. Watch-and-wait approach versus surgical resection after chemoradiotherapy for patients with rectal cancer (the OnCoRe project): a propensity-score matched cohort analysis. *Lancet Oncol.* **17**, 174–183 (2016). 26705854 10.1016/S1470-2045(15)00467-2

[8] Fernandez, L. M. et al. Conditional recurrence-free survival of clinical complete responders managed by watch and wait after neoadjuvant chemoradiotherapy for rectal cancer in the International Watch & Wait Database: a retrospective, international, multicentre registry study. *Lancet Oncol.* **22**, 43–50 (2021). 33316218 10.1016/S1470-2045(20)30557-X

[9] Custers, P. A. et al. Long-term Quality of Life and Functional Outcome of Patients With Rectal Cancer Following a Watch-and-Wait Approach. *JAMA Surg.* **158**, e230146 (2023). 36988922 10.1001/jamasurg.2023.0146 PMC10061319

[10] Smith, J. J. et al. Assessment of a Watch-and-Wait Strategy for Rectal Cancer in Patients With a Complete Response After Neoadjuvant Therapy. *JAMA Oncol.* **5**, e185896 (2019). 30629084 10.1001/jamaoncol.2018.5896 PMC6459120

[11] Garcia-Aguilar, J. et al. Organ Preservation in Patients With Rectal Adenocarcinoma Treated With Total Neoadjuvant Therapy. *J. Clin. Oncol.* **40**, 2546–2556 (2022). 35483010 10.1200/JCO.22.00032 PMC9362876

[12] Verheij, F. S. et al. Long-Term Results of Organ Preservation in Patients With Rectal Adenocarcinoma Treated With Total Neoadjuvant Therapy: The Randomized Phase II OPRA Trial. *J. Clin. Oncol.* **42**, 500–506 (2024). 37883738 10.1200/JCO.23.01208 PMC11578087

[13] National Comprehensive Cancer Network®. NCCN Clinical Practice Guidelines in Oncology: Rectal Cancer (Version 2.2024). Retrieved from https://www.nccn.org/guidelines/guidelines-detail?category=1&id=1461 (2024). 10.6004/jnccn.2024.0040 39151455

[14] de Jong, E. A., ten Berge, J. C. E. M., Dwarkasing, R. S., Rijkers, A. P. & van Eijck, C. H. J. The accuracy of MRI, endorectal ultrasonography, and computed tomography in predicting the response of locally advanced rectal cancer after preoperative therapy: A metaanalysis. *Surgery* **159**, 688–699 (2016). 26619929 10.1016/j.surg.2015.10.019

[15] Lee, K. C. et al. Current trends in nonoperative management for rectal adenocarcinoma: An unequal playing field?. *J. Surg. Oncol.* **126**, 1504–1511 (2022). 36056914 10.1002/jso.27082

[16] Zhou, J. et al. Serial Circulating Tumor DNA in Predicting and Monitoring the Effect of Neoadjuvant Chemoradiotherapy in Patients with Rectal Cancer: A Prospective Multicenter Study. *Clin. Cancer Res.* **27**, 301–310 (2021). 33046514 10.1158/1078-0432.CCR-20-2299

[17] Tie, J. et al. Serial circulating tumour DNA analysis during multimodality treatment of locally advanced rectal cancer: a prospective biomarker study. *Gut* **68**, 663–671 (2019). 29420226 10.1136/gutjnl-2017-315852 PMC6265124

[18] Wang, Y. et al. Utility of ctDNA in predicting response to neoadjuvant chemoradiotherapy and prognosis assessment in locally advanced rectal cancer: A prospective cohort study. *PLoS Med.* **18**, e1003741 (2021). 34464382 10.1371/journal.pmed.1003741 PMC8407540

[19] Liu, W. et al. Response prediction and risk stratification of patients with rectal cancer after neoadjuvant therapy through an analysis of circulating tumour DNA. *EBioMedicine* **78**, 103945 (2022). 35306340 10.1016/j.ebiom.2022.103945 PMC8933829

[20] Khakoo, S. et al. MRI Tumor Regression Grade and Circulating Tumor DNA as Complementary Tools to Assess Response and Guide Therapy Adaptation in Rectal Cancer. *Clin. Cancer Res.* **26**, 183–192 (2020). 31852830 10.1158/1078-0432.CCR-19-1996

[21] Vidal, J. et al. Clinical Impact of Presurgery Circulating Tumor DNA after Total Neoadjuvant Treatment in Locally Advanced Rectal Cancer: A Biomarker Study from the GEMCAD 1402 Trial. *Clin. Cancer Res.* **27**, 2890–2898 (2021). 33727257 10.1158/1078-0432.CCR-20-4769

[22] Manca, P. et al. Variant allele frequency in baseline circulating tumour DNA to measure tumour burden and to stratify outcomes in patients with RAS wild-type metastatic colorectal cancer: a translational objective of the Valentino study. *Br. J. Cancer* **126**, 449–455 (2022). 34811502 10.1038/s41416-021-01591-8 PMC8810873

[23] Bitterman, D. S. et al. Predictors of Complete Response and Disease Recurrence Following Chemoradiation for Rectal Cancer. *Front. Oncol.* **5**, 286 (2015). 26734570 10.3389/fonc.2015.00286 PMC4686647

[24] Amatu, A. et al. 509O Total neoadjuvant treatment (TNT) with non-operative management (NOM) for proficient mismatch repair locally advanced rectal cancer (pMMR LARC): First results of NO-CUT trial. *Ann. Oncol.* **35**, S431–S432 (2024).

[25] Watanabe, J. et al. Circulating tumor DNA for predicting complete response to total neoadjuvant therapy in locally advanced rectal cancer: ENSEMBLE-2. *J. Clin. Oncol.* **43**, 284–284 (2025).

[26] Zviran, A. et al. Genome-wide cell-free DNA mutational integration enables ultra-sensitive cancer monitoring. *Nat. Med.* **26**, 1114–1124 (2020). 32483360 10.1038/s41591-020-0915-3 PMC8108131

[27] Widman, A. J. et al. Ultrasensitive plasma-based monitoring of tumor burden using machine-learning-guided signal enrichment. *Nat. Med.* **30**, 1655–1666 (2024). 38877116 10.1038/s41591-024-03040-4 PMC7616143

[28] Frydendahl, A. et al. Detection of circulating tumor DNA by tumor-informed whole-genome sequencing enables prediction of recurrence in stage III colorectal cancer patients. *Eur. J. Cancer* **211**, 114314 (2024). 39316995 10.1016/j.ejca.2024.114314

[29] Nordentoft, I. et al. Whole-genome mutational analysis for tumor-informed detection of circulating tumor DNA in patients with urothelial carcinoma. *Eur. Urol.* **86**, 301–311 (2024). 38811314 10.1016/j.eururo.2024.05.014

[30] Alexandrov, L. B. et al. The repertoire of mutational signatures in human cancer. *Nature* **578**, 94–101 (2020). 32025018 10.1038/s41586-020-1943-3 PMC7054213

[31] Georgeson, P. et al. Genotoxic colibactin mutational signature in colorectal cancer is associated with clinicopathological features, specific genomic alterations and better survival. *medRxiv*10.1101/2023.03.10.23287127 (2024).

[32] Chang, L. et al. Prognostic Value of ctDNA Detection in Patients With Locally Advanced Rectal Cancer Undergoing Neoadjuvant Chemoradiotherapy: A Systematic Review and Meta-analysis. *Oncologist* **28**, e1198–e1208 (2023). 37294663 10.1093/oncolo/oyad151 PMC10712909

[33] Jothy, S. et al. Field effect of human colon carcinoma on normal mucosa: relevance of carcinoembryonic antigen expression. *Tumour Biol.* **17**, 58–64 (1996). 7501974 10.1159/000217967

[34] Kasi, P. M. et al. Circulating tumor DNA (ctDNA) for informing adjuvant chemotherapy (ACT) in stage II/III colorectal cancer (CRC): Interim analysis of BESPOKE CRC study. *J. Clin. Orthod.* **42**, 9–9 (2024).

[35] Kotani, D. et al. Molecular residual disease and efficacy of adjuvant chemotherapy in patients with colorectal cancer. *Nat. Med.* **29**, 127–134 (2023). 36646802 10.1038/s41591-022-02115-4 PMC9873552

[36] Nakamura, Y. et al. ctDNA-based molecular residual disease and survival in resectable colorectal cancer. *Nat. Med.* **1**, 12 (2024). 10.1038/s41591-024-03254-6 PMC11564113 39284954

[37] Parikh, A. R. et al. Minimal Residual Disease Detection using a Plasma-only Circulating Tumor DNA Assay in Patients with Colorectal Cancer. *Clin. Cancer Res.* **27**, 5586–5594 (2021). 33926918 10.1158/1078-0432.CCR-21-0410 PMC8530842

[38] Tie, J. et al. Circulating tumor DNA analysis informing adjuvant chemotherapy in locally advanced rectal cancer: The randomized AGITG DYNAMIC-Rectal study. *J. Clin. Orthod.* **42**, 12–12 (2024).

[39] Anagnostou, V. & Velculescu, V. E. Pushing the Boundaries of Liquid Biopsies for Early Precision Intervention. *Cancer Discov.* **14**, 615–619 (2024). 38571422 10.1158/2159-8290.CD-24-0037

[40] Tenaillon, O., Skurnik, D., Picard, B. & Denamur, E. The population genetics of commensal Escherichia coli. *Nat. Rev. Microbiol.* **8**, 207–217 (2010). 20157339 10.1038/nrmicro2298

[41] Nougayrède, J.-P. et al. Escherichia coli induces DNA double-strand breaks in eukaryotic cells. *Science* **313**, 848–851 (2006). 16902142 10.1126/science.1127059

[42] Pleguezuelos-Manzano, C. et al. Mutational signature in colorectal cancer caused by genotoxic pks+ E. coli. *Nature* **580**, 269–273 (2020). 32106218 10.1038/s41586-020-2080-8 PMC8142898

[43] Dziubańska-Kusibab, P. J. et al. Colibactin DNA-damage signature indicates mutational impact in colorectal cancer. *Nat. Med.* **26**, 1063–1069 (2020). 32483361 10.1038/s41591-020-0908-2

[44] Islam, S. M. A. et al. Uncovering novel mutational signatures by de novo extraction with SigProfilerExtractor. *Cell Genom* **2**, 10.1016/j.xgen.2022.100179 (2022). 10.1016/j.xgen.2022.100179 PMC9646490 36388765

[45] Sondka, Z. et al. COSMIC: a curated database of somatic variants and clinical data for cancer. *Nucleic Acids Res.* **52**, D1210–D1217 (2024). 38183204 10.1093/nar/gkad986 PMC10767972

