# Supplementary material for: Ultrasensitive ctDNA monitoring for organ preservation in patients with locally advanced rectal cancer
Source: NPJ Precis Oncol. 2025 Dec 11;10:8. doi: 10.1038/s41698-025-01208-w (PMC12775025; doi:10.1038/s41698-025-01208-w)
Supplement: Supplementary file 1 — Supplementary_Materials. [file 41698_2025_1208_MOESM1_ESM.pdf]

Ultrasensitive ctDNA monitoring for organ preservation in patients with locally advanced rectal cancer

Supplementary Materials

<u>Supplementary Figures:</u>

1. **CONSORT diagram.**
2. **Outcomes of the two TNT strategies.**
3. **ROC curves for TF and scCR prediction.**
4. **Isolated ctDNA assessment of P1 and P2.**
5. **Detailed P1-P2 ctDNA dynamic.**
6. **Post-restaging ctDNA dynamic.**
7. **CEA and TF oscillations in selected patients.**

<u>Supplementary Tables:</u>

1. **Relevant baseline clinical features depending on the neoadjuvant treatment arm.**
2. **Baseline clinical features and P0 ctDNA assessment.**
3. **scCR prediction with baseline features.**
4. **scCR prediction with P1 and P2 TF timepoints.**
5. **DFS prediction with P1 and P2 TF timepoints**

Supplementary Figures

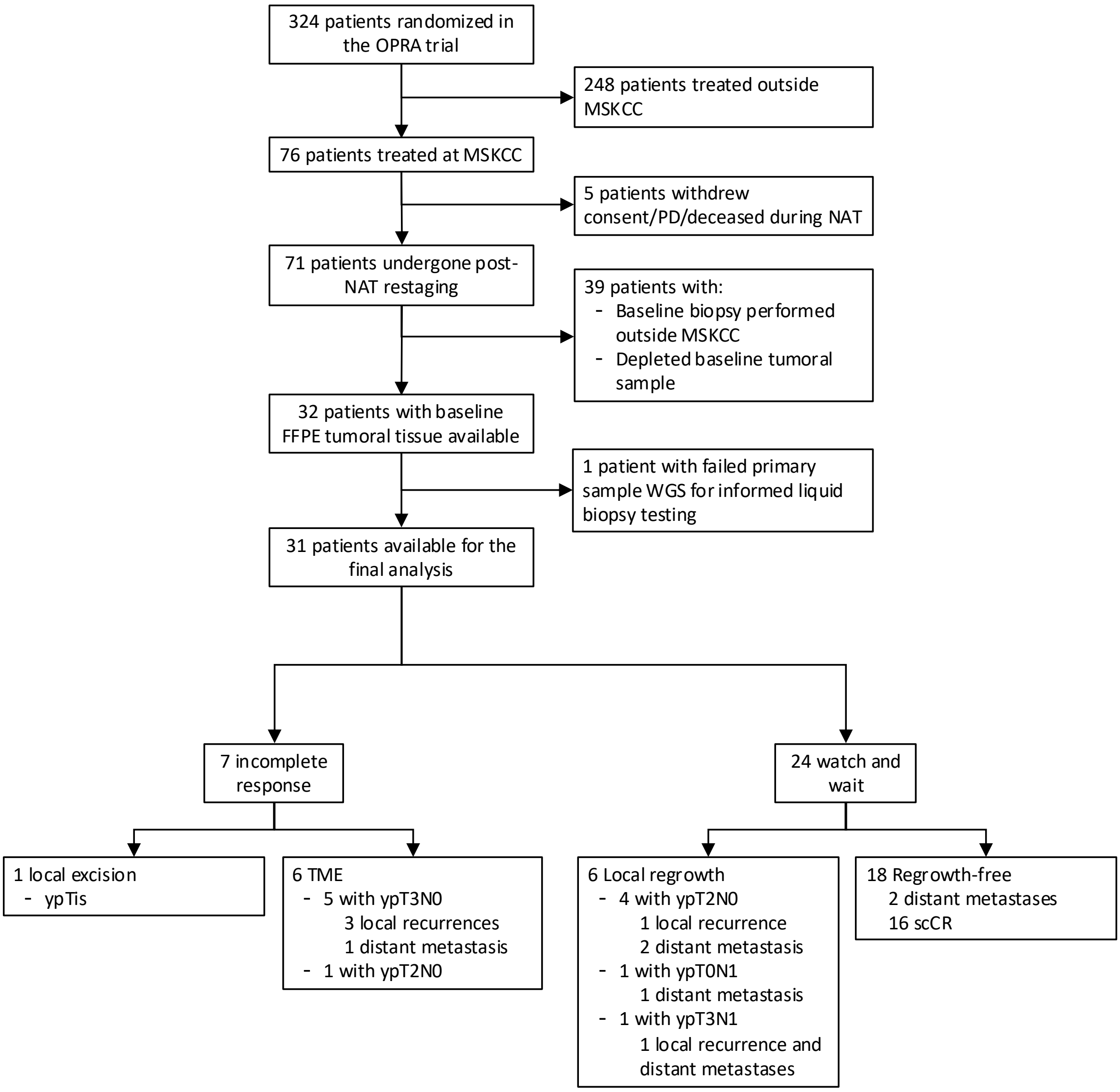

Supplementary Figure 1: CONSORT diagram. *CRT-CNCT: chemoradiation followed by consolidation chemotherapy; FFPE: formalin-fixed paraffin-embedded; INCT-CRT, induction chemotherapy followed by chemoradiation; MSKCC: Memorial Sloan Kettering Cancer Center; PD: progressive disease; NAT: neoadjuvant treatment; WGS: whole genome sequencing. cT: clinical tumor classification.*

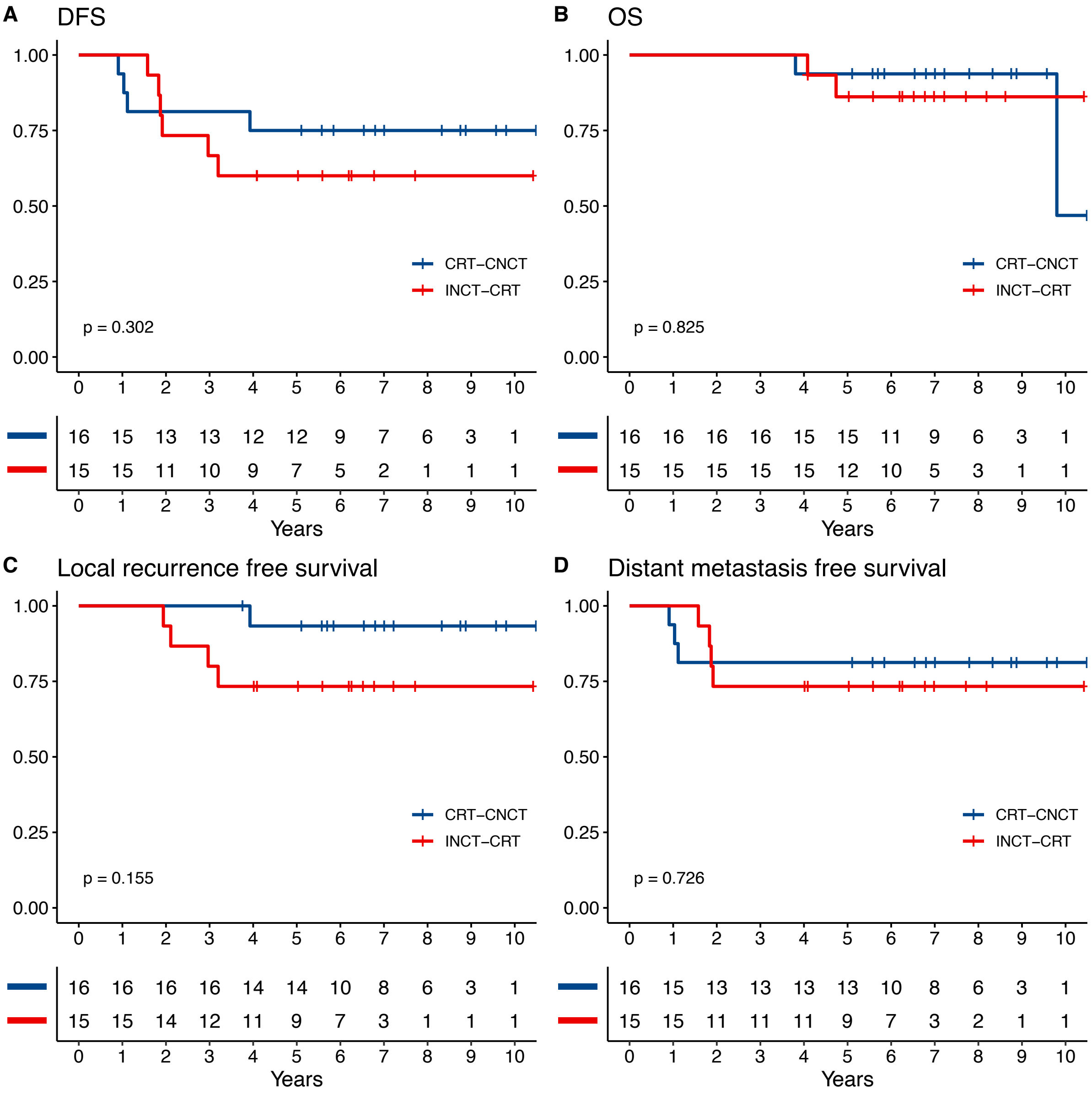

Supplementary Figure 2: Outcomes of the two TNT strategies. Kaplan Meier curves depicting, depending on the neoadjuvant treatment arm, the **A)** DFS, **B)** OS, **C)** local recurrence free survival and **D)** distant metastasis free survival. All outcomes are measured from the time of randomization. Reported p-values are calculated with Cox regression. *CRT-CNCT: chemoradiation followed by consolidation chemotherapy; DFS: disease-free survival; INCT-CRT, induction chemotherapy followed by chemoradiation; OS: overall survival.*

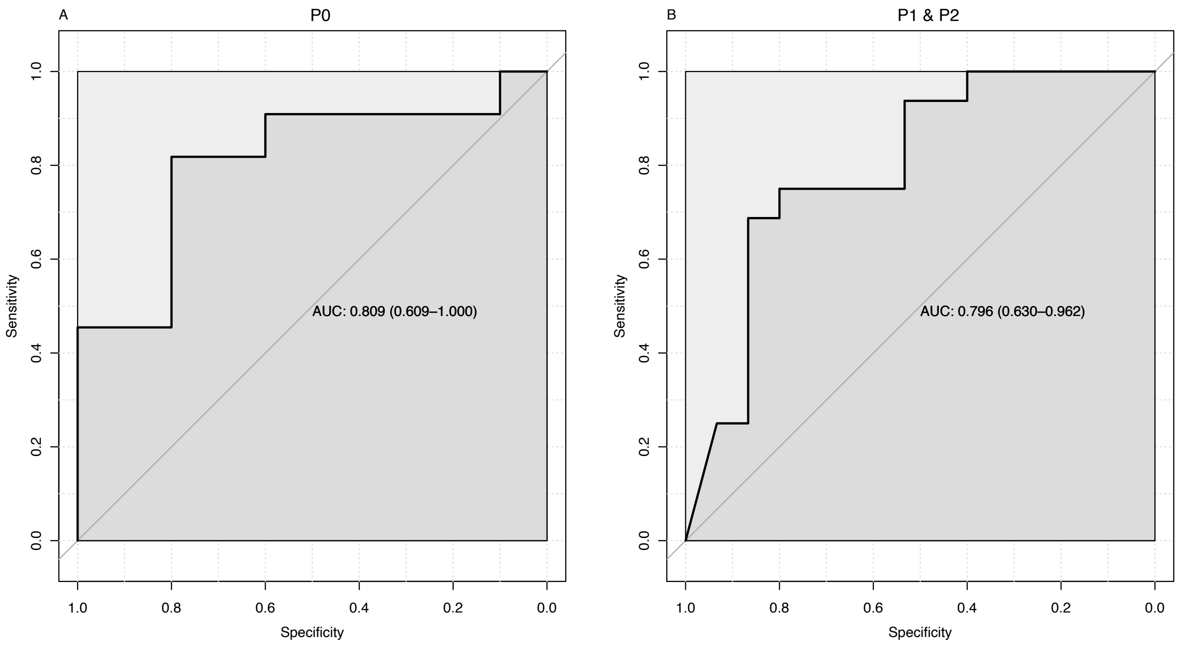

Supplementary Figure 3: ROC curves for TF and scCR prediction. ROC curves depicting specificity and sensitivity for scCR prediction using ctDNA tumor fraction at **A)** P0 and **B)** P1 and P2. *ctDNA: circulating tumor DNA; scCR: sustained clinical complete response.*

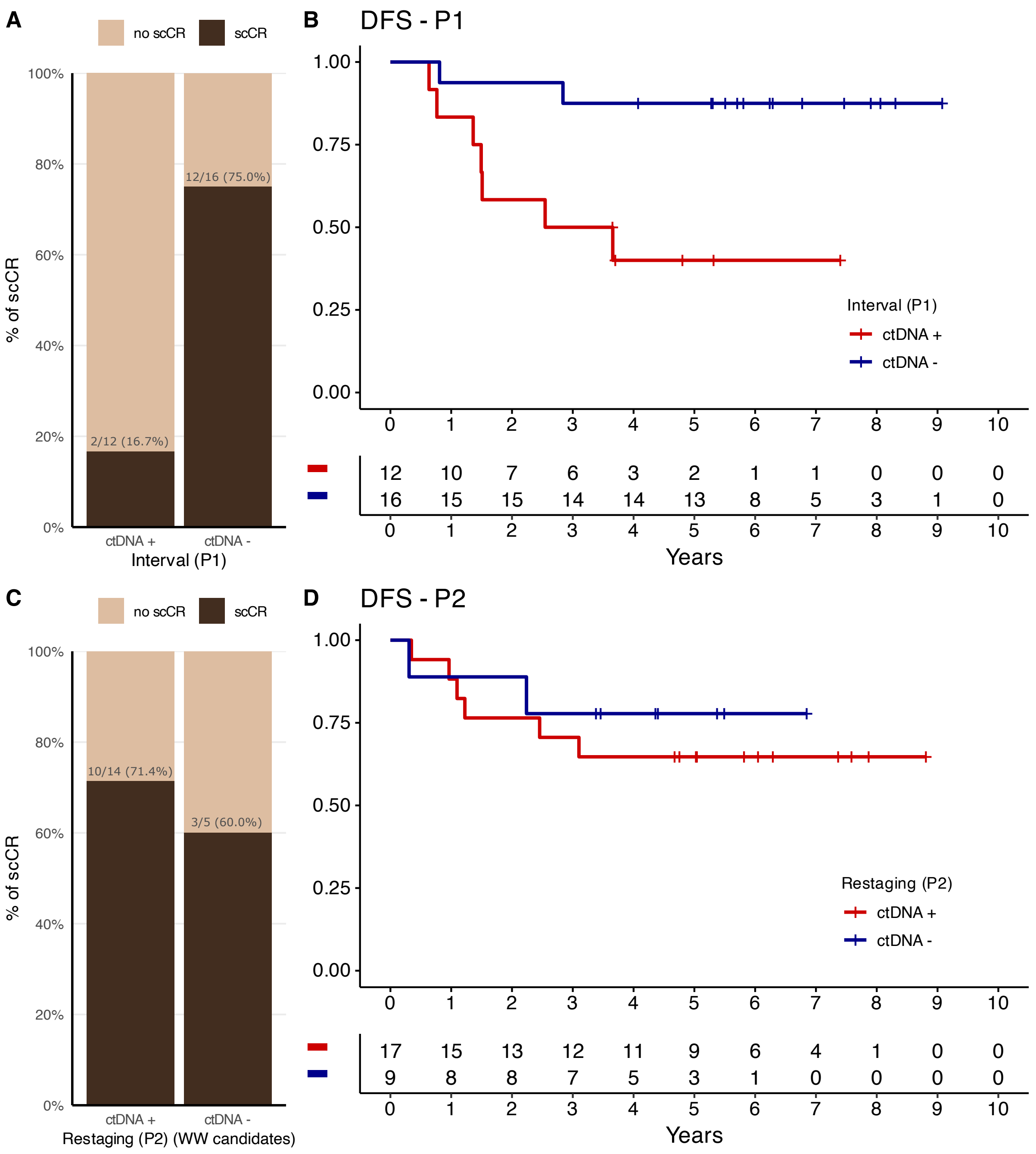

Supplementary Figure 4: Isolated ctDNA assessment of P1 and P2. A and **C)** Proportion of patients with scCR depending on the detection of ctDNA at, respectively, the P1 and the P2 timepoints; **B** and **D)** Kaplan-Meier curves depicting DFS depending on the detection of ctDNA at, respectively, the P1 and the P2 timepoints; DFS was measured starting from the moment of the liquid biopsy blood withdrawal. *ctDNA: circulating tumor DNA; DFS: disease-free survival; scCR: sustained clinical complete response.*

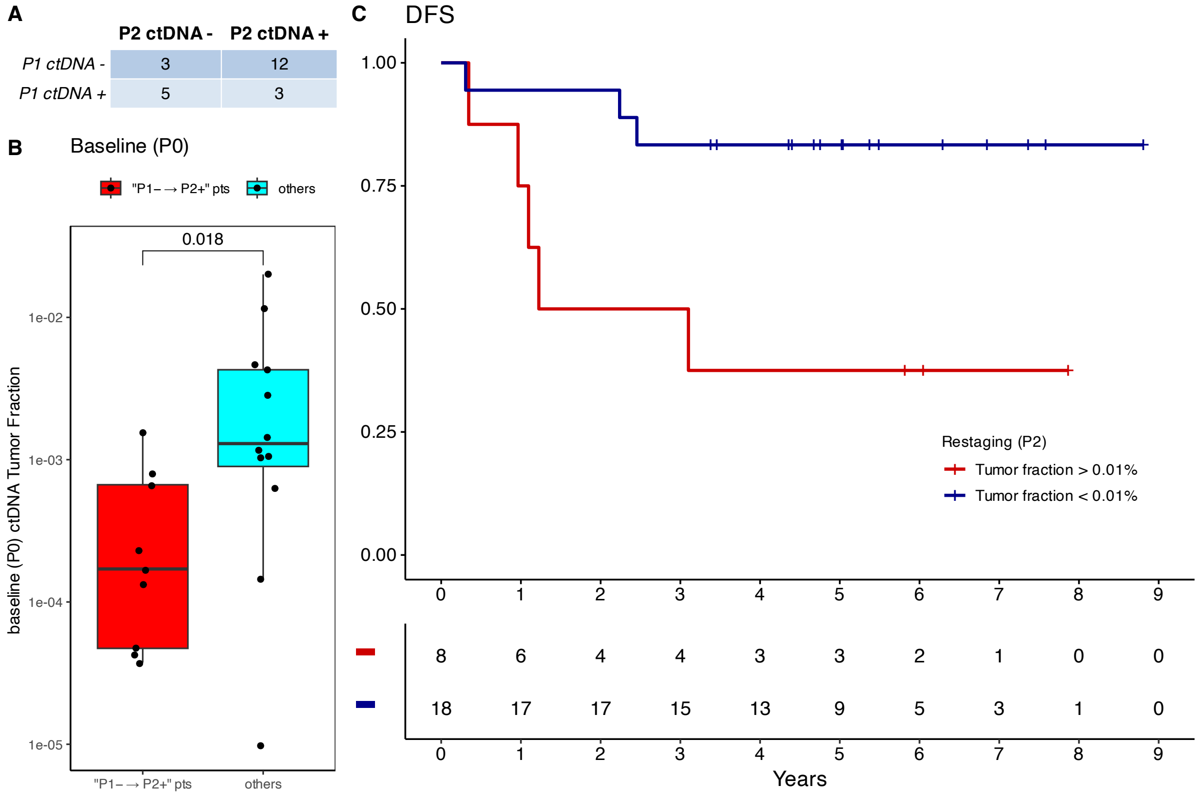

Supplementary Figure 5: Detailed P1-P2 ctDNA dynamic. A) table depicting the number of patients with a positive circulating tumor DNA (ctDNA) assessment at the P1 and the P2 timepoints for the 23 patients who had both a P1 and P2 liquid biopsy available. **B)** boxplot depicting the tumor fraction (TF) of the baseline P0 liquid biopsy: nine patients with negative P1 ctDNA assessment and positive P2 ctDNA assessment are represented in red and while the other 12 patients with a baseline P0 liquid biopsy available are represented in blue; the p-value was calculated using Wilcoxon signed-rank test; for graphic purposes, a sample with undetected ctDNA was plotted as TF=0.0001%. **C)** Kaplan Meier curves depicting DFS depending on the presence of a TF>0.01% at the P2 timepoint. *ctDNA: circulating tumor DNA; DFS: disease-free survival.*

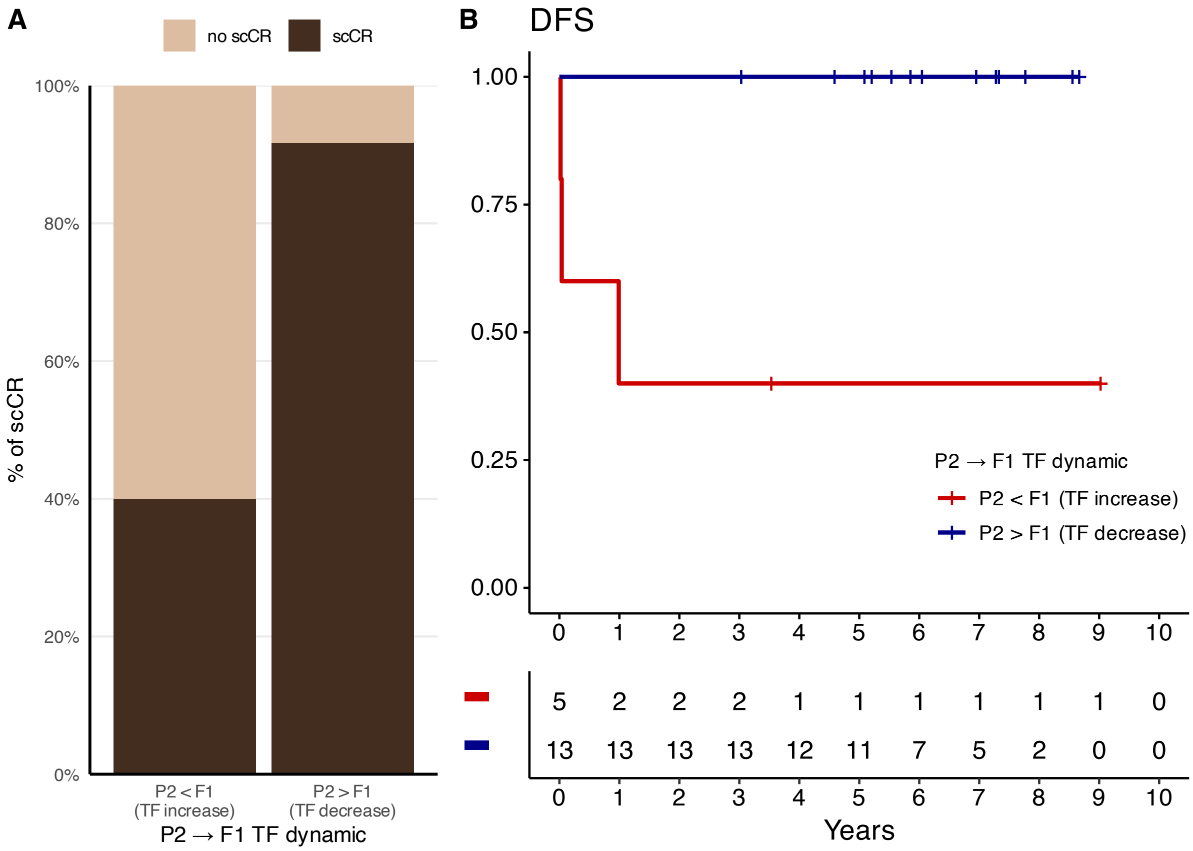

Supplementary Figure 6: Post-restaging ctDNA dynamic. **A)** barplot depicting the percentage of patients with a scCR depending on the TF dynamic between the P2 and the F1 timepoints. **B)** Kaplan Meier curves depicting DFS depending on the TF dynamic between the P2 and the F1 timepoints. *ctDNA: circulating tumor DNA; DFS: disease-free survival; TF: tumor fraction.*

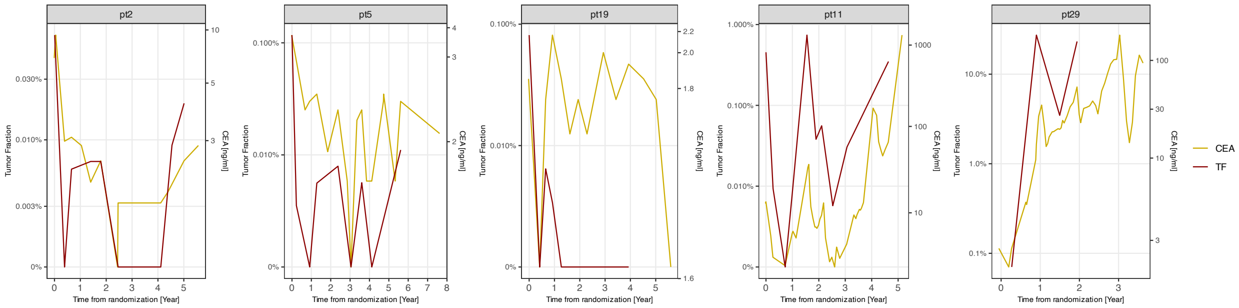

Supplementary Figure 7: CEA and TF oscillations in selected patients. CEA and TF represented over time for five selected patients: three patients without disease relapse (patient #2, patient #5 and patient #19) and two patients who experienced disease relapse (patient #11 and patient #29); the y scale on the left represents TF and the y scale on the right represents CEA in ng/ml; both axes are scaled to $\log_{10}$.

Supplementary Tables

Features		CRT-CNCT n=16 (51.6%)	INCT-CRT n=15 (48.4%)	p value
Age at diagnosis	Median (IQR)	51.7 (50.8 - 66.1)	58.5 (49.5 - 66.9)	0.874
Sex	Female	7 (43.8)	3 (20.0)	0.252
	Male	9 (56.2)	12 (80.0)	
Race	Asian	1 (6.2)	2 (13.3)	1.000
	Black or African American	1 (6.2)	0 (0.0)	
	White	14 (87.5)	13 (86.7)	
cT	1/2	4 (25.0)	0 (0.0)	0.101
	3/4	12 (75.0)	15 (100.0)	
cN	cN-	3 (18.8)	4 (26.7)	0.685
	cN+	13 (81.2)	11 (73.3)	
Anorectal verge distance [cm]	Median (IQR)	4.5 (4.0 - 5.8)	4.7 (3.9 - 5.8)	0.937
Primary tumor size [cm]	Median (IQR)	4.0 (2.8 - 5.1)	3.7 (3.2 - 4.2)	0.782
Extramural venous invasion	no	16 (100.0)	11 (73.3)	0.043
	yes	0 (0.0)	4 (26.7)	
Microsatellite instability	MSS	15 (100.0)	14 (100.0)	NA
	(missing)	1	1	

Supplementary Table 1: Relevant baseline clinical features depending on the neoadjuvant treatment arm. P-values are calculated using Fisher exact test for categorical variables or Wilcoxon signed-rank test for numerical variables. *cN: clinical nodal classification; CRT-CNCT: chemoradiation followed by consolidation chemotherapy; cT: clinical tumor classification; INCT-CRT, chemotherapy followed by chemoradiation.*

Feature		Median % TF (IQR)	p value
Age at diagnosis [years]	41 - 74	0.081 (0.014 to 0.158)	0.422
Sex	Female	0.019 (0.004 to 0.105)	0.128
	Male	0.108 (0.062 to 0.283)	
Race	Asian	0.130 (0.124 to 0.137)	0.269
	White	0.064 (0.013 to 0.145)	
Anorectal verge distance [cm]	0.0 - 13.4	0.081 (0.014 to 0.158)	0.795
Primary tumor size [cm]	1.9 - 8.5	0.081 (0.014 to 0.158)	<0.001
Extramural venous invasion	No	0.067 (0.013 to 0.283)	1.000
	Yes	0.091 (0.065 to 0.112)	
cN	cN-	0.018 (0.006 to 0.052)	0.014
	cN+	0.117 (0.049 to 0.352)	
cT	1/2	0.004 (0.004 to 0.005)	0.077
	3/4	0.101 (0.020 to 0.220)	
WGD	No	0.110 (0.021 to 0.317)	0.374
	Yes	0.074 (0.006 to 0.137)	
TMB [mut/Mb]	0.8 - 4.5	0.081 (0.014 to 0.158)	0.265
Ploidy	1.9 -3.9	0.081 (0.014 to 0.158)	0.069
CEA [µg/L]	0.9 – 22.6	0.081 (0.014 to 0.158)	0.248

Supplementary Table 2: Baseline clinical features and P0 ctDNA assessment. Key clinical and genetic features associated with the baseline P0 TF. reported p-values are calculated with Wilcoxon signed-rank test for categorical covariate and with Spearman correlation for numerical covariate. *cN: clinical nodal classification; cT: clinical tumor classification; TMB: tumor mutational burden; TF: tumor fraction; WGD: whole genome doubling.*

Feature		no scCR n (%)	scCR n (%)	OR (95% CI)	p-value
TF at P0*	-	-	-	0.23 (0.04-0.81)	0.048
Anorectal verge distance [cm]	-	-	-	0.77 (0.39-1.16)	0. 316
Age at diagnosis [year]	-	-	-	1.08 (0.99-1.21)	0.115
Sex	Female	3 (37.5)	5 (62.5)	-	
	Male	7 (53.8)	6 (46.2)	0.51 (0.08-3.04)	0.469
Race	Asian	1 (50.0)	1 (50.0)	-	
	Black or African American	1 (100.0)		NA#	NA#
	White	8 (44.4)	10 (55.6)	1.25 (0.04-35.07)	0.881
cT	1/2		2 (100.0)	-	-
	3/4	10 (52.6)	9 (47.4)	NA#	NA#
cN	cN-		6 (100.0)	-	-
	cN+	10 (66.7)	5 (33.3)	NA#	NA#
Primary tumor size [cm]	-	-	-	0.57 (0.22-1.09)	0.144
Extramural venous invasion	no	7 (41.2)	10 (58.8)	-	-
	yes	3 (75.0)	1 (25.0)	0.23 (0.01-2.26)	0.246
CEA [µg/L]	-	-	-	0.97 (0.80-1.15)	0.703

**TF was on a log_{10} scale; the patient with TF=0 was considered as having a TF of 0.001%*
#not possible to compute a Odds Ratio

Supplementary Table 3: scCR prediction with baseline features. Univariate logistic regression for scCR prediction. *cN: clinical nodal classification; cT: clinical tumor classification; scCR: sustained clinical complete response; TF: tumor fraction.*

Feature		no scCR n (%)	scCR n (%)	OR (95% CI)	p-value
Maximum TF at P1 or P2*	-	-	-	0.10 (0.01-0.48)	0.018
Anorectal verge distance [cm]	-	-	-	0.68 (0.39-1.00, p=0.099)	0. 316
Age at diagnosis [year]	-	-	-	1.05 (0.98-1.14, p=0.147)	0.115
Sex	Female	4 (40.0)	6 (60.0)	-	-
	Male	11 (52.4)	10 (47.6)	0.61 (0.12-2.76)	0.521
cT	1/2	1 (25.0)	3 (75.0)	-	-
	3/4	14 (51.9)	13 (48.1)	0.31 (0.01-2.77)	0.335
cN	cN-	1 (14.3)	6 (85.7)	-	-
	cN+	14 (58.3)	10 (41.7)	0.12 (0.01-0.85)	0.066
Primary tumor size [cm]	-	-	-	0.75 (0.39-1.27)	0.307
Extramural venous invasion	no	12 (44.4)	15 (55.6)	-	-
	yes	3 (75.0)	1 (25.0)	0.27 (0.01-2.39)	0.278
CEA [µg/L]	-	-	-	0.97 (0.86-1.05)	0.447

**TF was on a log_{10} scale; patients with TF=0 was considered as having a TF of 0.001%*

Supplementary Table 4: scCR prediction with P1 and P2 TF timepoints. Univariate logistic regression for scCR prediction. *cN: clinical nodal classification; cT: clinical tumor classification; scCR: sustained clinical complete response; TF: tumor fraction.*

Feature		HR (95% CI)	p-value
Maximum TF at P1 or P2*	-	4.20 (1.87-9.43)	0.001
Anorectal verge distance [cm]	-	1.02 (0.79-1.33)	0. 861
Age at diagnosis [year]	-	0.96 (0.91-1.03)	0. 254
Sex	Female	-	-
	Male	1.18 (0.31-4.58)	0. 806
cT	1/2	-	-
	3/4	1.28 (0.16-10.12)	0. 814
cN	cN-	-	-
	cN+	NA#	NA#
Primary tumor size [cm]	-	1.42 (0.87-2.33)	0. 159
Extramural venous invasion	no	-	-
	yes	1.80 (0.38-8.52)	0. 461
CEA [µg/L]	-	1.03 (0.98-1.07)	0. 254

**TF was on a log_{10} scale; patients with TF=0 was considered as having a TF of 0.001%*
#not possible to compute a Hazard Ratio

Supplementary Table 5: DFS prediction with P1 and P2 TF timepoints. Univariate cox regression for DFS prediction. *cN: clinical nodal classification; cT: clinical tumor classification; TF: tumor fraction.*
